# Supplementary material for: Scaf1 promotes respiratory supercomplexes and metabolic efficiency in zebrafish
Source: EMBO Rep. 2020 Jun 4;21(7):e50287. doi: 10.15252/embr.202050287 (PMC7332985; doi:10.15252/embr.202050287)
Supplement: Supplementary file 1 — Appendix [file EMBR-21-e50287-s001.docx]

Appendix.

**Scaf1 mediates respiratory supercomplexes and promotes metabolic efficiency in zebrafish**

CONTENT:

[Appendix figure S1. Generation of Scaf1 loss-of-function zebrafish models. 2](#_Toc38042098)

[Appendix figure S2. The Scaf1 loss-of-function phenotype is stable in adulthood, recessive and not maternally contributed. 4](#_Toc38042099)

[Appendix table S1. Nutritional content of Sparos high fat (HFD), control and low proteins/low fats (LP/LF) diets. 5](#_Toc38042100)


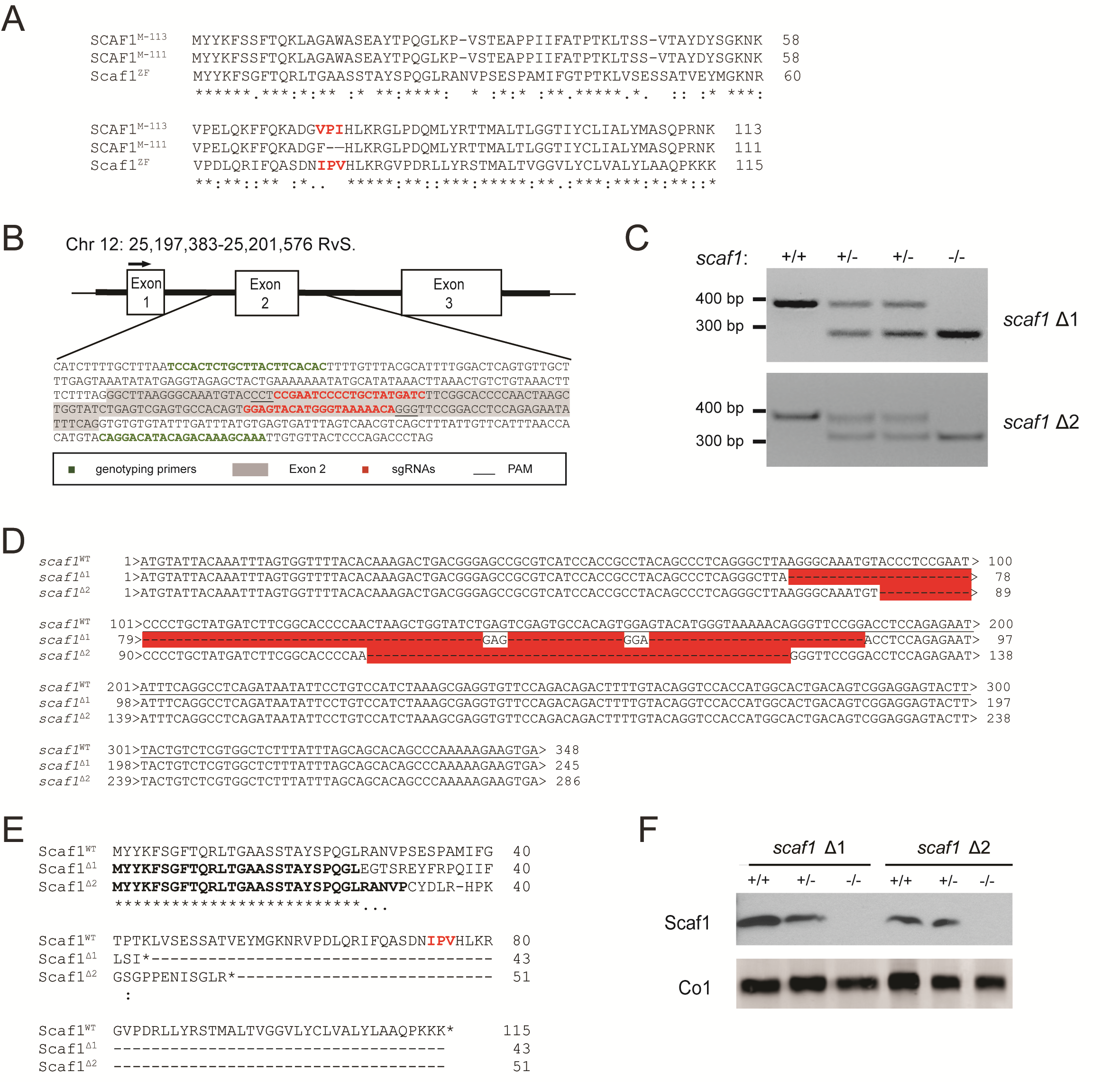


**Appendix figure S1. Generation of Scaf1 loss-of-function zebrafish models. A**, Alignment of the amino acid sequences of mouse SCAF1^113^, SCAF1^111^ and zebrafish Scaf1 (also known as Cox7a2l, Cox7a3). **B**, CRISPR/Cas9 target design for generation of zebrafish Scaf1 loss-of function-models. In green, primer sequences for genotyping, in red sgRNAs with their PAM sequence underlined. Exon sequences are highlighted in grey. **C**, Representative image of PCR products of the two established Scaf1 loss-of-function lines (*scaf1*^Δ1^ and *scaf1*^Δ2^). **D**, Sequence of *scaf1*^Δ1^ and *scaf1*^Δ2^ mutant alleles. In red, the deleted sequence. **E**, Predicted amino acid sequence of *scaf1*^Δ1^ and *scaf1*^Δ2^. **F**, Western blot of isolated mitochondria from *scaf1*^Δ1^ and *scaf1*^Δ2^ fish (representative of 4 biological replicates).


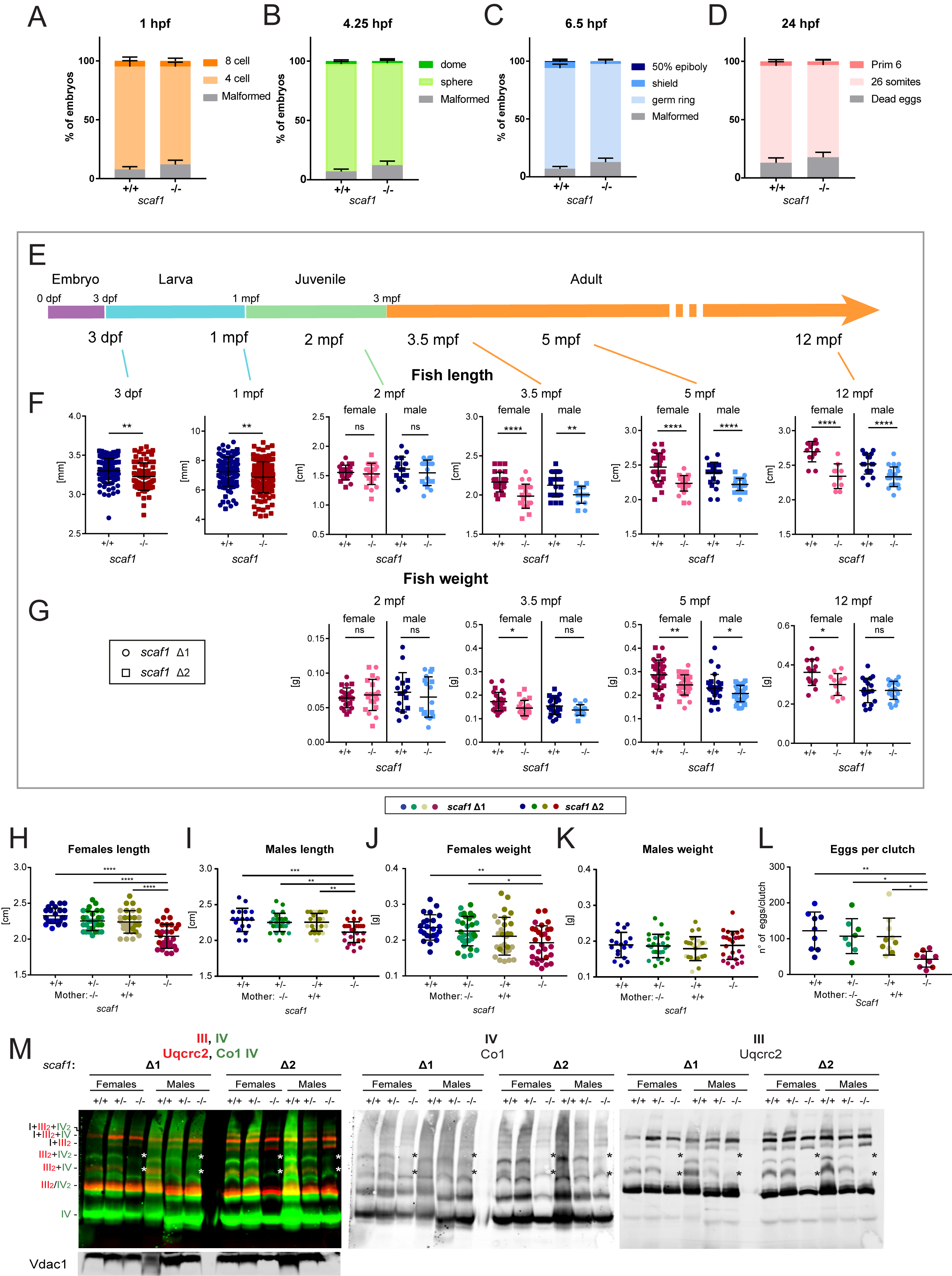


**Appendix figure S2. The Scaf1 loss-of-function phenotype is stable in adulthood, recessive and not maternally contributed.** **A-D**, Percentage of *scaf1*^+/+^ and *scaf1*^-/-^ fish (Δ1 n=5, Δ2 n=5 clutches of around 60-200 animals each). No significant difference (unpaired t-test p>0.05) was found between *scaf1*^+/+^ and *scaf1*^-/-^. **E-G,** *scaf1*^-/-^ fish size at different developmental stages. **E,** Timeline of zebrafish development. **F**, Fish length and **G**, weight at the indicated time point: 3 dpf (Δ1 n=40, Δ2 n= 55), 1 mpf (Δ1 n=60, Δ2 n= 65), 2 mpf (females Δ1 n=11, Δ2 n= 12, males Δ1 n=8, Δ2 n= 8), 3.5 mpf (females Δ1 n=10, Δ2 n= 10, males Δ1 n=8, Δ2 n= 8), 5 mpf (females Δ1 n=15, Δ2 n= 16, males Δ1 n=13, Δ2 n= 23), 12 mpf (females Δ1 n=10-13, males Δ1 n=18-23). **H-M**, Size at 5 mpf of heterozygous offspring coming from a cross where the mother was *scaf1*^+/+^ and *scaf1*^-/-^. Length of F1 **H**, females and **I**, males and weight of **J**, females(WT1 +/+ n=7, Δ1 +/- n=16, Δ1 -/+ n=17, Δ1 -/- n=16, WT2 +/+ n=15, Δ2 +/- n=12, Δ2 -/+ n=11, Δ2 -/- n=12) and **K**, males (WT1 +/+ n=7, Δ1 +/- n=12, Δ1 -/+ n=10, Δ1 -/- n=11, WT2 +/+ n=10, Δ2 +/- n=12, Δ2 -/+ n=14, Δ2 -/- n=13). **L**, number of eggs per clutch (WT1 +/+ n=5, Δ1 +/- n=4, Δ1 -/+ n=6, Δ1 -/-, n=6, WT2 +/+ n=4, Δ2 +/- n=4, Δ2 -/+ n=4, Δ2 -/- n=3). **M**, BNGE and immunoblot analysis with the indicated antibodies comparing the impact of Scaf1 deletion in Δ1 and Δ2 fish lines. Asterisks indicate absent bands. Split channels are included. **E-G**, Unpaired t-test. **H-L**, One-way ANOVA. Data are represented as mean ± SD. * p<0.05, ** p<0.01, *** p<0.001, **** p<0.0001.

| **Diet composition** | **HFD** | **CONTROL** | **LP/LF** |
| --- | --- | --- | --- |
| Crude protein, % feed | 55 | 55 | 27.8 |
| Crude fat, % feed | 30.1 | 14.1 | 7 |
| Fiber, % feed | 1.6 | 17.2 | 50.9 |
| Ash, % feed | 6.7 | 6.7 | 5.5 |
| Others % (aminoacids, vit, metals...) | 6.7 | 7 | 8.7 |
| **Gross Energy, MJ/kg feed** | 22.2 | 18.3 | 16 |
| **Gross Energy Kcal/kg feed** | 5302.4 | 4370.9 | 3821.5 |
| **Gross Energy of protein and fat Kcal/kg feed** | **4904.3 (141%)** | **3464.3 (100%)** | **1748.06 (50%)** |

# **Appendix table S1. Nutritional content of Sparos high fat (HFD), control and low proteins/low fats (LP/LF) diets.**
