# Supplementary material for: Scaf1 promotes respiratory supercomplexes and metabolic efficiency in zebrafish
Source: EMBO Rep. 2020 Jun 4;21(7):e50287. doi: 10.15252/embr.202050287 (PMC7332985; doi:10.15252/embr.202050287)
Supplement: Supplementary file 2 — Expanded View Figures PDF [file EMBR-21-e50287-s002.pdf]

Expanded View Figures

Figure EV1. Characterization of OXPHOS super-assembly in zebrafish.

A–C Split channels of BNGE of mouse (M) C57BL/6J (111), CD1(113), and zebrafish (ZF) muscle mitochondria shown in Fig 1A and B.

D, E Immunodetection of the indicated proteins after 2D BNGE/DDM electrophoresis of whole-body zebrafish mitochondria (representative of n = 3). Merged (D) and (E) split channels.

© 2020 The Authors

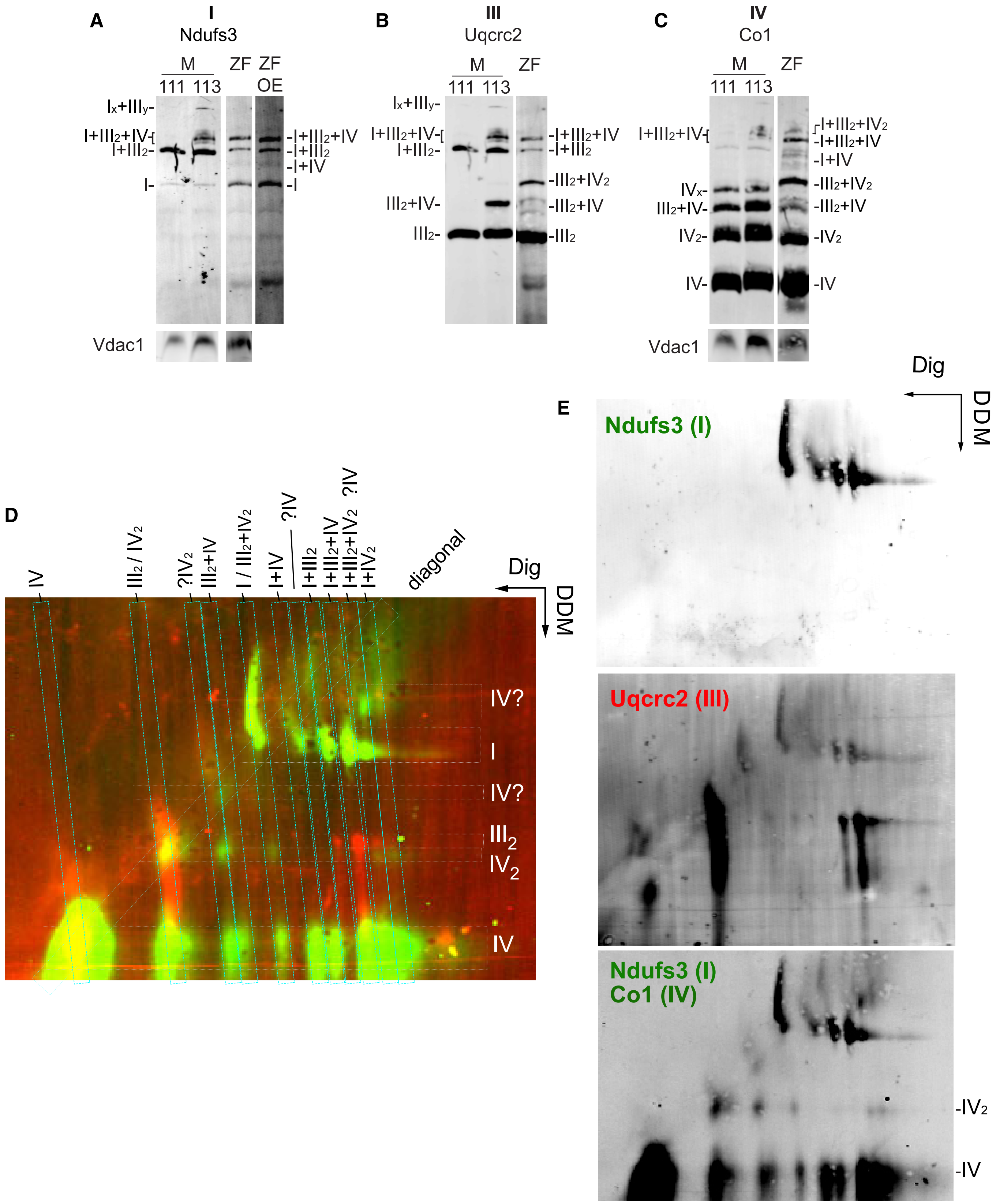

Figure EV1.

 © 2020 The Authors

Figure EV2. OXPHOS super-assembly in whole zebrafish and mouse liver in homeostasis and low-protein/low-fat diet.

A–E BNGE of mouse (M) C57BL/6J (111) and CD1(113) liver mitochondria and zebrafish (ZF) whole-body mitochondria, digitonin-solubilized. (A–C) Immunodetection of the indicated proteins, (D) CI and (E) CIV in-gel activity (representative of two technical and three biological replicates).

F Immunodetection of the indicated proteins of digitonin-solubilized whole-body zebrafish mitochondria with different concentrations of digitonin.

G–I BNGE of zebrafish fed with low-protein/low-fat diet (LP/LF) and control diet (CD). (G) variation of BMI of fish after 6 weeks in LP/LF and CD (H) Representative images. (I) Representative BNGE of whole fish mitochondria of fish fed during 6 weeks in the indicated diet (experimental replicates n = 2 are composed by a pool of n = 2 biological replicates).

© 2020 The Authors

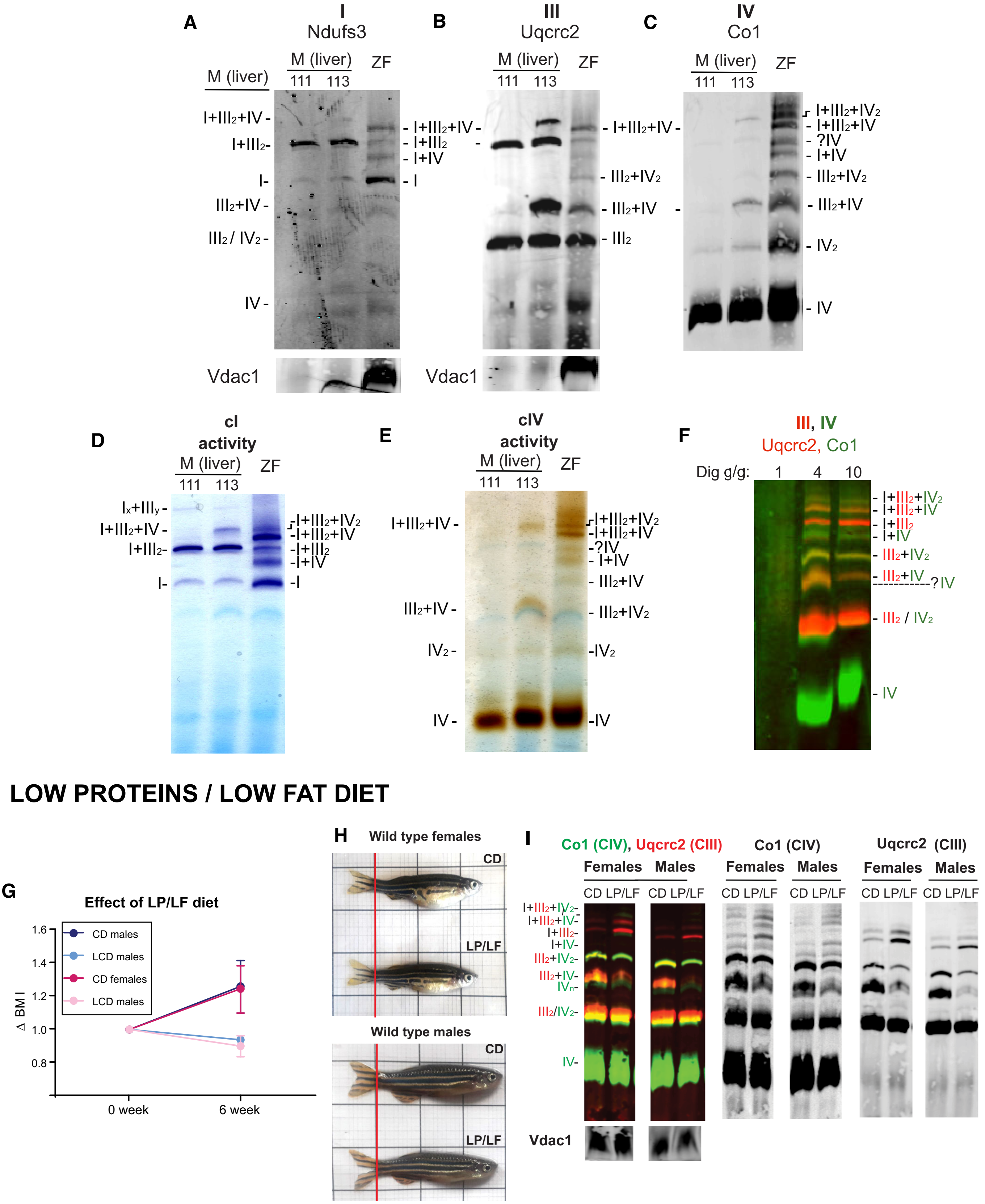

Figure EV2.

 © 2020 The Authors

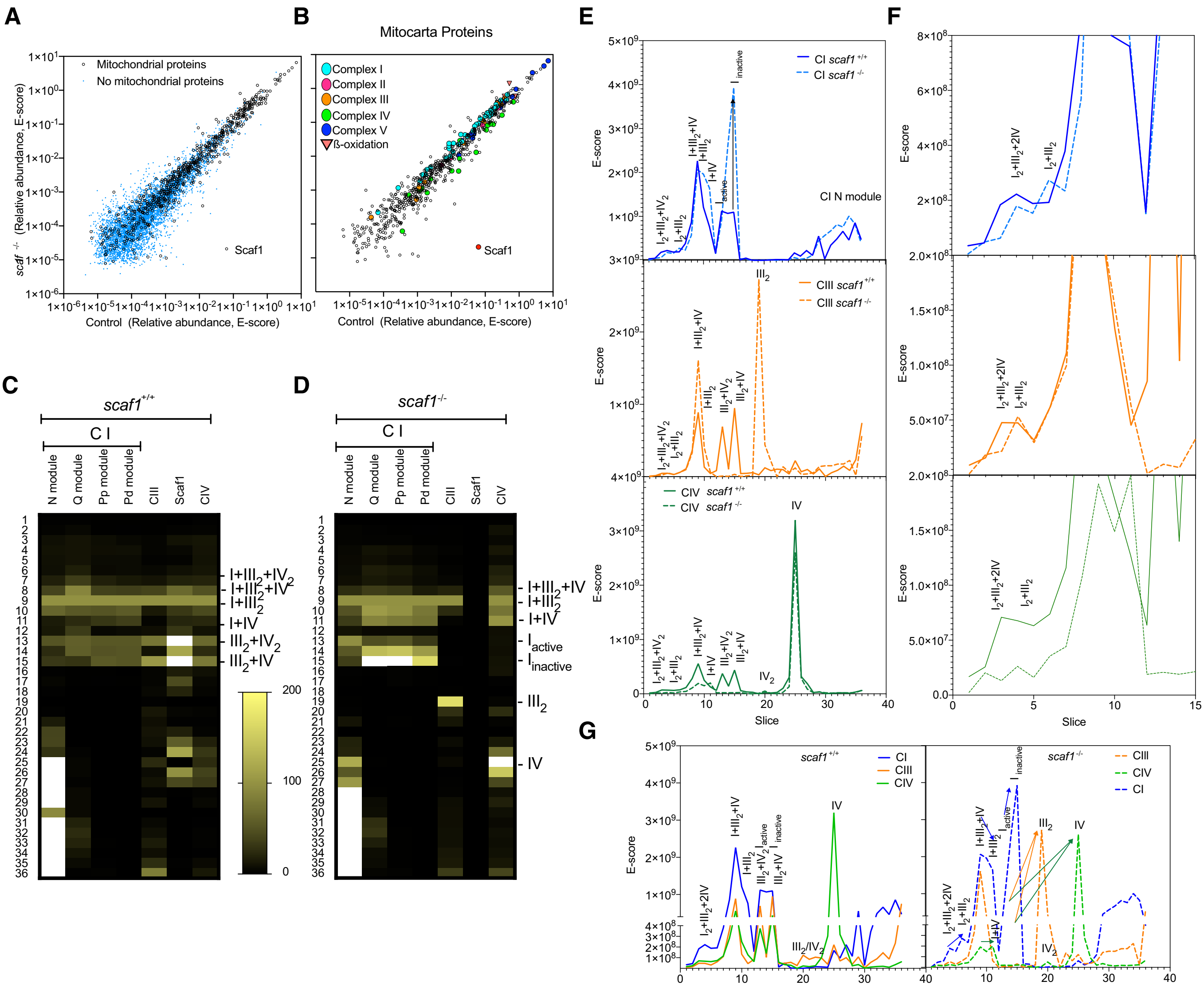

Figure EV3. Analysis of mitochondrial complexes by Blue-DiS proteomics.

A, B Correlation between the abundance of proteins (expressed as sum of *E*-scores of the corresponding peptides) detected in the analysis of *scaf1*$^{+/+}$ and *scaf1*$^{-/-}$ animals. Proteins were considered mitochondrial according to the classification in the mouse MitoCarta 2.0 database. Non-mitochondrial proteins include true non-mitochondrial proteins and potential mitochondrial proteins that failed to be identified as such. In (B), only the mitochondrial proteins are represented, indicating the proteins from the indicated groups.

C, D Heatmaps showing the summed absolute abundance of selected protein groups across BNGE gel slices. For a better comparison, absolute abundances were normalized using the values of slice 9 as a reference. Qualitative migration of the added *E*-score value for each indicated complex, subcomplex, or protein. For each line, data were normalized within a 100–0 range, with 100 being the value of slice 9. The color scale is established as a linear increase from black (being 0) to the green in slice 9 (being 100). Any value over 100 is white.

E–G Analysis of the quantitative differences between Blue-DiS profiles of *scaf1*$^{+/+}$ and *scaf1*$^{-/-}$ animals. Differences in quantity profiles of CI-, CIII-, and CIV-related complexes and SCs in (E) control and (F) *scaf1*$^{-/-}$ samples. The insets focus on the differences at very high molecular weights (slices 1–15). (G) Comparative analysis of quantitative profiles of complexes and SCs in control or *scaf1*$^{-/-}$ samples. Arrows indicate increase, decrease, or shifts of complexes observed between *scaf1*$^{-/-}$ and control samples.

© 2020 The Authors

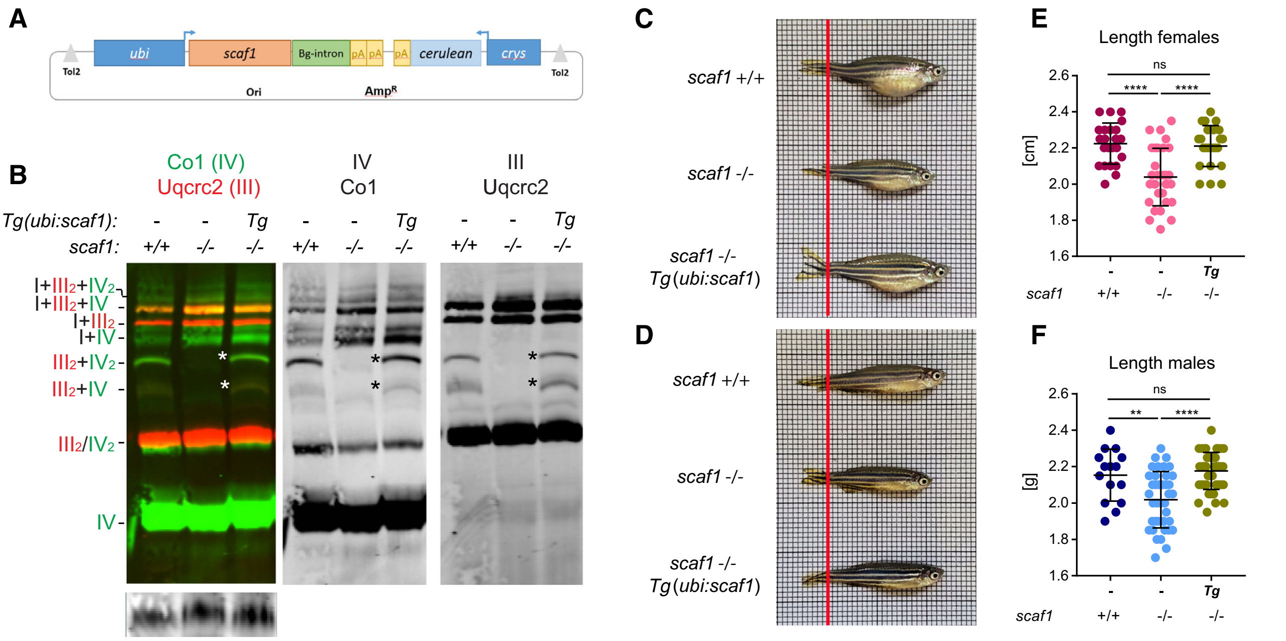

Figure EV4. Transgenic expression of *scaf1* recovers CIII and CIV super-assembly and fish size of *scaf1*$^{-/-}$ fish.

A Scheme of the transgenic construct *Tg*(*ubi:scaf1*). ubi, ubiquitin promoter. Bg-intron, beta-globin intron, pA, poly A, *crys*, crystalline promoter (used as selection marker).

B Immunodetection of the indicated proteins of BNGE from whole fish mitochondria *scaf1*$^{\Delta1/\Delta1}$ (–/–) expressing the transgenic construct *Tg*(*ubi:scaf1*) in heterozygosis, their *scaf1*$^{\Delta1/\Delta1}$ siblings with no transgenic expression, and *scaf1*$^{+/+}$ zebrafish (representative BNGE of *n* = 5). Asterisks mark bands' absence in *scaf1*$^{\Delta1/\Delta1}$ (–/–) recovered by the transgenic expression of *scaf1*.

C, D Representative images from *scaf1*$^{+/+}$, *scaf1*$^{\Delta1/\Delta1}$ (*scaf1*$^{-/-}$), and scaf1$^{-/-}$, Tg/– (C) female and (D) male zebrafish (3 mpf).

E, F Size of *scaf1*$^{+/+}$, *scaf1*$^{\Delta1/\Delta1}$ (*scaf1*$^{-/-}$), and scaf1$^{-/-, \, Tg/-}$ fish (E) length of females (*scaf1*$^{+/+}$ *n* = 23, *scaf1*$^{-/-}$ *n* = 39 and *scaf1*$^{-/-, \, Tg/-}$ *n* = 25); and (F) males (*scaf1*$^{+/+}$ *n* = 15, *scaf1*$^{-/-}$ *n* = 31 and scaf1$^{-/-, \, Tg/-}$ *n* = 44).

Data information: One-way ANOVA. ns $P > 0.05$, $^{**}P < 0.01$, $^{****}P < 0.0001$. Data are represented as mean ± SD.

 © 2020 The Authors

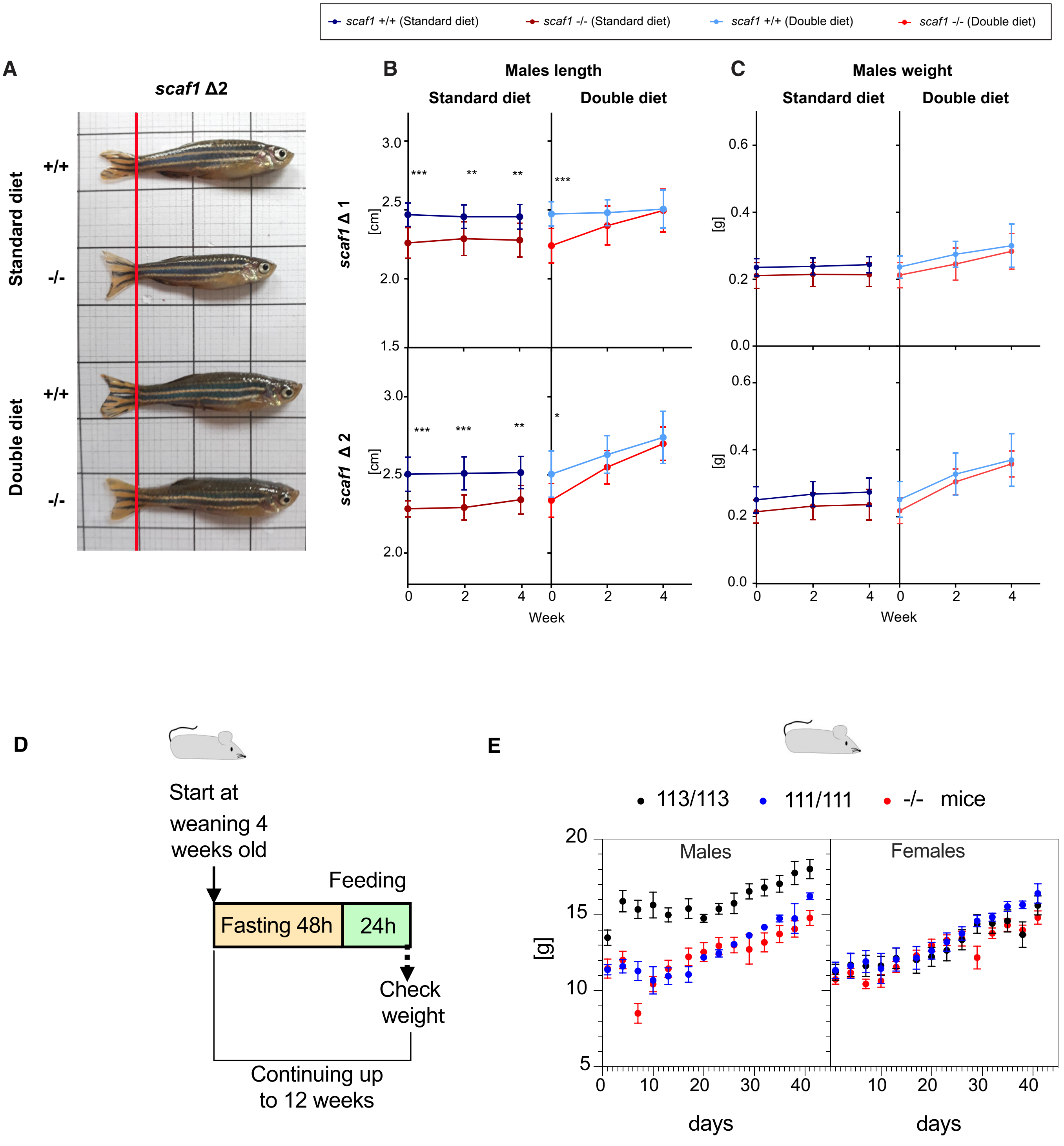

Figure EV5. Diet-induced recovery of *scaf1*$^{-/-}$ phenotypes in males and diet effect in SCAF1-deficient mice.

A Representative images of *scaf1*$^{-/-}$ and *scaf1*$^{+/+}$ males fed with the indicated diets.

B, C Size of males after the indicated diet. (B) Changes in length and (C) weight over time (Δ1 +/+ n = 10, Δ1 −/− n = 10, Δ2 +/+ n = 10, Δ2 −/− n = 7–8).

D, E Effect of SCAF1 loss of function on weight gain in mice after starvation. (D) Scheme of the food restriction experiment in mice. (E) Impact of food restriction in C57BL/6JOlaHsd mice with the functional version of SCAF1 113/113, with the spontaneous mutation in SCAF1 111/111 (natural C57BL/6JOlaHsd mice harbor a non-functional version of SCAF1) and in C57BL/6JOlaHsd mice without SCAF1 (SCAF1 KO, −/−). Males 111/111 n = 3; males KO n = 6; males 113/113 n = 2; females 111/111 n = 5; females KO n = 8; females 113/113 n = 5.

Data information: (B, C) Two-way ANOVA. Data are represented as mean ± SD. *P < 0.05, **P < 0.01, ***P < 0.001. (E) Data are represented as mean ± SEM.

© 2020 The Authors
